# The temporal window of visual processing throughout adulthood

**DOI:** 10.3389/fnins.2025.1547959

**Published:** 2025-03-20

**Authors:** Xianghang He, Xiaowei Ruan, Menglu Shen, Junli Yuan, Cheng Li, Yan Yang, Jinli Zhu, Rong Cui, Zhong-Lin Lu, Jiang-Fan Chen, Fang Hou

**Affiliations:** ^1^The Eye and Brain Center, State Key Laboratory of Ophthalmology, Optometry and Vision Science, Eye Hospital, Wenzhou Medical University, Wenzhou, Zhejiang, China; ^2^National Engineering Research Center of Ophthalmology and Optometry, Eye Hospital, Wenzhou Medical University, Wenzhou, China; ^3^School of Ophthalmology and Optometry and Eye Hospital, Wenzhou Medical University, Wenzhou, China; ^4^Rysm Bio Limited, Shenzhen, China; ^5^Division of Arts and Sciences, NYU Shanghai, Shanghai, China; ^6^Center for Neural Science and Department of Psychology, New York University, New York, NY, United States; ^7^NYU-ECNU Institute of Brain and Cognitive Science at NYU Shanghai, Shanghai, China

**Keywords:** aging, contrast threshold, the elaborated perceptual template model, external noise, temporal window, temporal deficits, integration masking

## Abstract

Aging is associated with declines in various visual functions, including visual processing in the temporal domain. However, how visual processing in the temporal domain changes throughout adulthood remains unclear. To address this, we recruited 30 adults aged 20 to 70 years. By systematically manipulating the stimulus onset asynchrony (SOA) of external noise masks, we measured contrast thresholds in an orientation discrimination task across five SOA conditions and one no mask condition. We hypothesized that the threshold would change with age, and that this change would depend on the SOA condition. Our results showed that thresholds increased with age at all SOA conditions, except for the no mask condition. To further explore temporal processing dynamics, we applied the elaborated perceptual template model to the contrast thresholds, which allowed us to extract the temporal processing window—describing how visual processing efficiency varies over time. The model provided a good fit to the data for all participants. We then extracted the peak and full width at half maximum (FWHH) of the processing window, reflecting the maximum efficiency and temporal extend of processing window, respectively, from the best-fit model for each participant. Regression analysis revealed that the peak decreased, while the FWHH increased with age, indicating that the temporal window of visual processing became wider and less efficient as age increased. Our cross-sectional study suggests that our ability to process dynamic visual information gradually declines with age in two significant ways: a decrease in peak efficiency and increased vulnerability to temporal disturbances.

## Introduction

As the number and proportion of people aged 60 years and older increase globally, aging poses significant challenges to both individuals and society. Understanding the structural and functional changes associated with aging is crucial, not only in the context of “pathological” aging but also for “healthy” aging. Notably, older adults exhibit declines in many visual functions (Owsley, 2011; Andersen, 2012; Owsley, 2016), which cannot be fully explained by changes in the optics of the eye (Weale, 1987; Bennett et al., 1999). Instead, these visual impairments are largely attributed to changes in central visual processing during aging (Roinishvili et al., 2011; Pilz et al., 2015; Agnew and Pilz, 2017; Saija et al., 2019).

As visual input constantly changes, human observers must quickly integrate instantaneous pieces of information across time to form a stable perception. Prior studies have shown that aging can affect visual temporal integration (Di Lollo et al., 1982; Saija et al., 2019; He et al., 2020). For instance, Di Lollo et al. (1982) presented their participants with two 5 × 5 dot matrices simultaneously and asked them to identify which one had a missing dot. Both matrices consisted of briefly and successively plotted dots. To perform the task, participants had to integrate the dots over time. Di Lollo et al. (1982) measured the critical interval required to achieve 75% accuracy and found that older participants needed a longer critical inter-dot interval than younger participants. Using visual rapid serial presentation, Saija et al. (2019) asked participants to identify targets (such as /, \, O, and their combinations) and evaluated the relative frequencies of integration reports, that is, reporting of a single response combining features of two targets. They found that older adults exhibited more integration than younger adults across all stimulus durations, especially at longer ones. Similar results were found in backward masking studies (Atchley and Hoffman, 2004; Roinishvili et al., 2011; Pilz et al., 2015; Agnew and Pilz, 2017), where researchers found that older participants needed a longer target-mask stimulus onset asynchrony (SOA) to achieve the same performance as younger participants. These findings suggest that older observers may have a longer temporal integration window.

**Figure 1:**
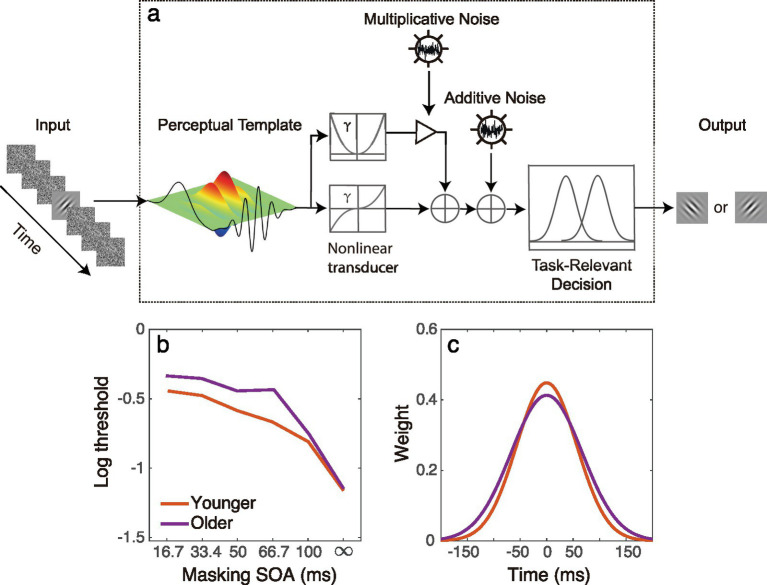
**(a)** Illustration of the ePTM using an orientation discrimination task. The model consists of a perceptual template, additive internal noise, multiplicative noise, a non-linear transducer function, and a decision unit. The visual input, a Gabor embedded in dynamic external noise, is first processed by the perceptual template with a specific temporal profile and then passes through a non-linear transducer. The process is affected by internal additive and multiplicative noise. Finally, a decision is made based on the noisy representation. **(b)** A representative threshold vs. masking SOA dataset of the young (red) and older people (purple) that can be fed into the ePTM to derive the temporal integration window. Each dataset consists of contrast thresholds at different SOAs. Noise masks at different SOA condition results in different masking effect (threshold elevation). The dynamic noise caused greater masking effect in the older group than in the younger group. **(c)** The temporal window extracted by the ePTM in older observers (purple) had a significantly lower peak and broader width compared to that in the younger observers (red).

In our recent study (He et al., 2020), we computationally modeled the longer temporal integration window observed in older adults using the elaborated perceptual template model (ePTM; Lu et al., 2004). The ePTM was originally developed to quantitatively characterize the full temporal window of visual attention (Lu et al., 2004) and has become a powerful tool to study temporal processing in multiple populations, such as patients with amblyopia, high myopia and older adults (He et al., 2020; Hu et al., 2021; Zheng et al., 2021). The model consists of a perceptual template, additive internal noise, multiplicative noise, a non-linear transducer function, and a decision unit (Figure 1a; Lu and Dosher, 1999; Lu et al., 2004; Lu and Dosher, 2008). In the study of He et al. (2020), we adopted the ePTM to investigate the full temporal window for visual processing in younger and older individuals, with stimulus visibility strictly controlled. We presented participants with a Gabor target temporally surrounded by dynamic noise masks, and we measured the contrast threshold at different target-mask intervals. We selected a low spatial frequency grating to ensure equal visibility for both younger and older groups. To model the contrast thresholds with ePTM, the visual input (i.e., a Gabor embedded in dynamic external noise), is first processed by the perceptual template with a specific temporal profile, and then passed through a non-linear transducer. The process is affected by internal additive and multiplicative noise. Finally, a decision is made based on the noisy representation. The ePTM analysis revealed that the temporal window in older observers had a significantly lower peak and broader width (Figures 1b,c), indicating that the visual system of older individuals is more susceptible to temporal disturbances (He et al., 2020).

However, most of the aforementioned studies focused on participants at the extremes of the age spectrum, leaving unclear how the temporal window in visual processing changes across adulthood. Understanding the full trajectory of the functional changes across adulthood is crucial. First, it can provide insight into the onset of these changes (Owsley et al., 1983; Tang and Zhou, 2009; Wang et al., 2009). Second, differing functional trajectories may offer valuable insights into the neurophysiological underpinnings of aging. For example, Bartzokis et al. (2010) measured myelin integrity in the frontal lobes and finger-tapping speed in participants aged 23 to 80 years. They found a significant correlation between myelin integrity in the frontal lobes and finger-tapping speed, with both sharing an indistinguishable lifespan trajectory, suggesting that maximum motor speed changes over a lifetime may depend on brain myelin integrity. Furthermore, data spanning a wide age range could help mitigate the potential for false positives caused by interindividual variability within small age ranges. Therefore, it is essential to measure the temporal processing window across multiple age groups.

In this study, we estimated the temporal window of visual processing in participants aged 20 to 70 years, using a similar experimental paradigm to the previous study (He et al., 2020). The participants performed a grating orientation discrimination task, and we measured contrast thresholds under different target-mask SOA conditions. We hypothesized that the threshold would change with age, and that this change would depend on the SOA condition. The ePTM (Figure 1) was also used to quantitatively estimate the temporal processing window across different age groups. Regression analyses were conducted to explore how the temporal window as well as other ePTM parameters such as internal additive noise and template gain, change with age.

## Methods

### Participants

This is a cross-sectional study. Thirty participants aged between 20 and 70 years were recruited from the campus of Wenzhou Medical University or local communities in Wenzhou, China. The participants were evenly distributed across each decade, with six participants in each decade group. All participants went through detailed ophthalmologic and optometric examinations conducted by the first and third authors (XH, MS), and all had normal or corrected-to-normal vision (minimal angle resolvable, MAR ≤ 1.0 arcmin). None of the participants had any eye diseases, diabetes, hypertension, mental illness, or cognitive deficits (Mini Mental State Examination, MMSE = 28.9 ± 0.57). A few older participants had minimal cataracts in one or both eyes, but these were not clinically significant and required no intervention, according to the Preferred Practice Pattern Guideline from the American Academy of Ophthalmology Preferred Practice Pattern Cataract/Anterior Segment Panel (Olson et al., 2017).

The study adhered to the tenets of the Declaration of Helsinki and was approved by the institutional review board of human subject research of the Eye hospital, Wenzhou Medical University (2020-111-K-98-01). All observers were naive to the purpose of the study. Written informed consent was obtained from each participant before the experiment.

### Sample size

The sample size was determined based on our previous research (He et al., 2020), where the effect sizes in data between the 20s and 60s age groups ranged from 0.19 to 0.47. Since ANOVA and linear regression are conceptually similar, we used the average effect size (0.27) to calculate the required sample size. Assuming a power of 0.8 and a significance level of 0.05, calculation suggested that 24 observers would be sufficient for regression analysis. Ultimately, we decided to include 30 participants for the current study. Additionally, we combined data from our previous study (He et al., 2020), which included participants in their 20s, 50s and 60s, with data from the current study. We analyzed this combined dataset in two ways: once with all 24 participants (total *N* = 54) and again only with 9 participants (total *N* = 39) whose ages were different from those in the current study. Both analyses yielded the same results, reinforcing the robustness of the trend in the temporal window change from 20 to 70 years.

### Apparatus

The experiment was conducted in a dimly lighted room with a PC computer (ProDesk 680 G2 MT, Hewlett Packard, Palo Alto, CA, United States). The program used in the experiment was coded in MATLAB (The Math Works Corp., Natick, MA, United States) with Psychtoolbox extensions (Kleiner et al., 2007). Stimuli were displayed on a gamma-corrected cathode-ray tube (CRT) display (Multiscan G520, Sony Corp., Tokyo, Japan). The display had a spatial resolution of 800 × 600 pixels and a refresh rate of 120 Hz. The mean luminance of the display was 44.6 cd/m^2^. Each pixel subtended 0.01 degrees at the viewing distance of 2.88 m.

A chin/forehead rest was used to minimize head movement during the experiment. Participants viewed the stimuli through their dominant eye with their best correction at the viewing distance if any. The non-dominant eye was occluded by an opaque patch. Eye dominance was assessed by the hole-in-card method (Dane and Dane, 2004).

### Stimuli

The stimulus was the same as that used in our previous studies (He et al., 2020). The target is a Gabor with spatial frequency of 2 cycles per degree (cpd). We carefully chose this spatial frequency to make sure that the target had similar visibility for observers at different ages, as the contrast sensitivity for stationary gratings at low spatial frequencies did not change through adulthood (Owsley et al., 1983; He et al., 2020). The Gabor stimuli subtended 300 × 300 pixels and oriented ±45° from vertical. The standard deviation of the Gabor was 0.5 degrees.

The external noise images also had a size of 300 × 300 pixels, consisted of noise elements with size of 10 × 10 pixels. The Weber contrast of each noise element was independently and identically sampled from a Gaussian distribution with mean zero and a standard deviation of 0.33. Background luminance was added to each external noise images.

**Figure 2:**
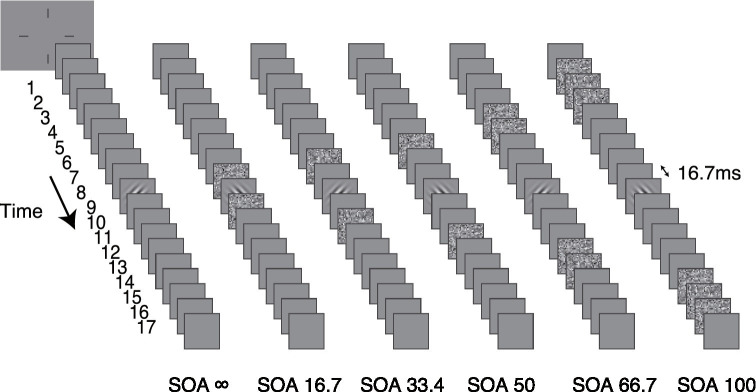
The stimuli in six different dynamic external noise configurations are shown. The external noise-target SOA was manipulated in different conditions. From left to right, they are SOA ∞ (no mask condition), SOA 16.7, SOA 33.4, SOA 50, SOA 66.7 and SOA 100, respectively.

The stimulus in each trial consisted of 17 sequential image frames and was presented at the center of the display (Figure 2). Each frame lasted two display refresh cycles (16.7 ms). The Gabor target appeared in the ninth frame. The external noise frames were placed symmetrically around the target frame in time. There were six conditions in the experiment: no mask (SOA ∞), and external noise image occupied the 8 and 10th frames (SOA 16.7), the 7 and 11th frames (SOA 33.4), the 6 and 12th frames (SOA 50), the 4, 5, 13, and 14th frames (SOA 66.7), and the 1st, 2nd, 3rd, 15, 16, and 17th frames (SOA 100), respectively. The remaining frames in the 17-frame sequence were filled with blank images. The external noise configuration was specifically designed to cover the entire temporal curve of “integration masking” (SOA ± 150 ms; Koenderink and van Doorn, 1980; Watson et al., 1986; Georgeson, 1987) within a reasonable test duration and without sacrificing precision. Because the temporal function of the masking effect is bell shaped—changing rapidly at short SOAs and slowly at long SOAs (Breitmeyer and Öğmen, 2006)—the external noise mask has a stronger effect at shorter SOAs and a weaker effect at longer SOAs. To ensure the threshold remained within a measurable range (i.e., not too low to detect), the external noise mask was set to a longer duration (multiple frames) for SOA conditions of 66.7 ms and 100 ms. In addition, since the temporal weight at long SOAs is relatively flat, the average temporal weight over multiple external noise frames still provides a good approximation of the “true” temporal profile in long SOA conditions.

### Design

Each experimental session consisted of six conditions, with an equal number of trials (100) for each condition, and the trials were mixed in random order. Therefore, one session consisted of 600 trials and took about 40 min to complete. The contrast threshold in each condition was estimated using a Bayesian adaptive procedure, the Psi method (Kontsevich and Tyler, 1999). Prior to the experimental session, participants completed a practice session of approximately 100 trials to ensure they fully understood the task.

### Procedure

All participants were given 5 mins to adapt to the dim test environment before the experiment started. Each trial began with a brief tone signaling its onset. A crosshair fixation (250 ms) was presented at the center of the screen, followed by blank screen (125 ms) with background luminance. Then the 17-frame (16.7 × 17 = 283.9 ms) stimulus sequence was presented and followed by another blank frame that lasted until response. Observers were required to identify whether the Gabor stimulus was –45° or + 45° relative to vertical by pressing the left or right arrow key on the computer keyboard. Auditory feedback was provided after each correct response. A new trial started 500 ms after the response.

### The elaborated perceptual template model

In the following, we provide a formal description of the ePTM. As shown in Figure 1, the visual input consists of the target Gabor with contrast *c* and external noise images with root mean square (RMS) contrast $N_{ext}$ . The input is first processed by the perceptual template, a spatiotemporal filter, which applies a total gain with value β to the target relative to the external noise (Lu and Dosher, 1999; Lu and Dosher, 2008), and a temporal weight $W_{t}$ at each time *t* for the external noise. The template gain *β* reflects the overall system efficiency when processing the signal relative to the external noise, while $W_{t}$ represents the relative gain of the system to the external noise at different times. The process was followed by a non-linear transducer characterized by a power function with exponent *γ*, which models the nonlinearity in visual processing. The process was also affected by internal additive noise $N_{a}$ and multiplicative noise $N_{m}$ , which simulate random intrinsic fluctuations of neural responses. When the input strength increases, *Na* does not change, while *Nm* increases. The effect of internal multiplicative noise is equivalent to that of contrast gain control (Dao et al., 2006). Finally, the noisy representation is sent to the decision unit. The signal-to-ratio $d^{\prime }$ of at the decision stage can be written as:


(1) $$d^{\prime }=\frac{{\left(\beta c\right)}^{\gamma }}{\sqrt{{N_{a}}^{2}+{\left({\left(\beta c\right)}^{2\gamma }+{N_{ext}}^{2\gamma }\right)}^{2}{N_{m}}^{2}+N_{ext}^{2}}}$$

For external noise images, each with variance $\sigma^{2}$ , the total variance of external noise in a given temporal configuration is


(2) $${N_{ext}}^{2}=\overset{17}{\underset{t=1}{\sum }}F_{t}{\left(W_{t}\sigma_{t}\right)}^{2}$$

where $\sigma_{t}=0.33$ , and $F_{t}=1$ when the noise frame is present, and $\sigma_{t}=0$ and $F_{t}=0$ when the blank frame is present (Figure 1). Since the total gain of the perceptual template to external noise is normalized to 1.0 in the PTM (Lu and Dosher, 1999), the temporal weights should satisfy the following constraint:


(3) $$\overset{17}{\underset{t=1}{\sum }}{W_{t}}^{2}=1$$

Recall that there are five different external noise configurations, so we can only obtain the average weight for the multi-frame conditions:


(4) $$W_{t}=\{\;\begin{array}{c} \;W_{16.7},\mathrm{if}\;t=8,10,\\ \;W_{33.4},\mathrm{if}\;t=7,11,\\ \;W_{50.0},\mathrm{if}\;t=6,12,\\ \;W_{66.7},\mathrm{if}\;t=4,5,13,14,\\ \;W_{100.0},\mathrm{if}\;t=1,2,3,15,16,17\text{,} \end{array}$$

By plugging Equations 2–4 into Equation 1, we can obtain the percent correct from the *d’* (Hacker and Ratcliff, 1979):


(5) $$P\left(c\right)=\overset{+\infty }{\underset{-\infty }{\int }}\phi \left(x-d^{\prime }\left(c,f\right)\right)\Phi^{m-1}\left(x\right)dx$$

where *m* = 2 for our orientation discrimination task, and ϕ (.) and Φ (.) are the probability density and cumulative probability density functions of a standard normal distribution. Equation 5 is used to account for response data. The ePTM had eight free parameters: *Na, Nm, β, γ, W*_16.7_*, W*_33.4_*, W*_50.0_ and *W*_66.7_. Under the constraint in Equation 2, *W*_100_ can be calculated from the other four weights.

### Analysis

For each observer, the threshold in each of the six conditions was estimated from the best-fitting Weibull psychometric function. A maximum likelihood procedure (Watson, 1979) was used to fit the ePTM to the trial-by-trial behavioral data. A *χ*^2^ test was used to examine the goodness of fit of the model for each participant (Watson, 1979),


$$\chi^{2}\left(df\right)=2\log\left(\frac{LLH_{\mathrm{baseline}}}{LLH_{\mathrm{model}}}\right)\text{,}$$

where *LLH*_baseline_ is the likelihood of a model that is the data itself (which serves as the baseline), *LLH*_model_ is the likelihood of the best-fit ePTM model, and df=n−k , with *n* being the number of data points, and *k* the number of model parameters. A *p* > 0.05 indicates that the ePTM prediction is statistically equivalent to the data, suggesting a good fit. The ePTM parameters were estimated from the best-fit model.

A mixed design ANCOVA, with within-subject factor SOA and between subject covariate age, was used to examine whether the threshold or temporal weight linearly depends on age and whether the threshold or temporal weight at different SOA conditions changed with age differently. The slopes (regression coefficient) were also estimated to represent the trend of change. A Student’s t-test was used to compare the slopes between two conditions (Andrade and Estévez-Pérez, 2014). To prevent the inflation of false positive rates due to the multiple comparisons, a max-*t* procedure was used to correct the *p*-value (Westfall and Young, 1993; Sandrine et al., 2003). The data were randomly permuted 10,000 times. In each permutation, the maximal statistic (*t*-value) was recorded across all the measures. After 10,000 permutations, a single empirical sampling distribution that described the maximal statistic across all measures was obtained to provide the distribution for the null hypothesis. Then, for each measure in the unpermuted set, the *p*-value was determined according to this distribution. The resulting *p*-values were corrected for family-wise error. The data and Matlab code used for analyses are available through the link https://osf.io/sy5cv/?view_only=aa547c58887d42fca973821b77762fb3.

## Results

### Masking effects

**Figure 3:**
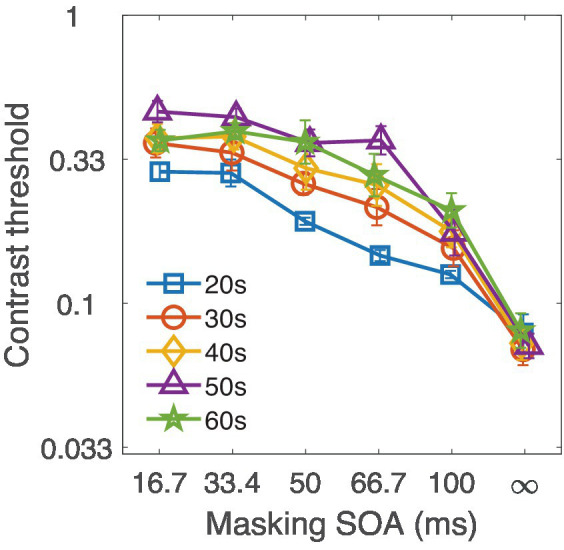
Contrast thresholds for each decade group are shown as a function of SOA. Different colors represent the various age groups.

The contrast threshold of 2AFC grating discrimination task is plotted as a function of SOA for each decade group in Figure 3. As shown in the figure, threshold increased as external noise mask became closer (i.e., as SOA decreased) to the target, indicating stronger interference. Moreover, this masking effect became more pronounced with increasing age, while the threshold at SOA ∞ (without external noise mask) remained nearly the same for all age groups.

A mixed-design ANCOVA with the factor SOA and covariate age was conducted to investigate how age affected the threshold at different SOAs. There was a significant effect of SOA [*F*(5, 140) = 193.1, *p* = 5.65 × 10^−61^], and age has also significantly affected thresholds [*F*(1, 28) = 15.26, *p* = 0.001], suggesting a linear relationship between threshold and age. In addition, there was a significant interaction between age and SOA [*F*(5, 140) = 3.631, *p* = 0.004], indicating that thresholds at different SOAs changed with age in distinct ways.

**Figure 4:**
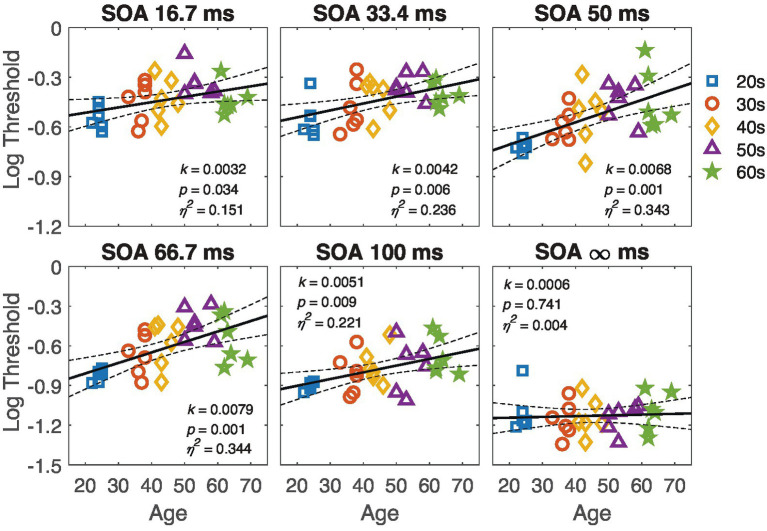
The contrast thresholds at different SOA are plotted against age in the respective panels. The black line represents the regression line, with dashed lines indicating the 95% confidence interval. The slope *k*, p-value, and effect size *η*^2^ are listed in the corresponding panels. Each marker represents individual data points, and different colors denote different age groups.

Regression analysis revealed that thresholds at SOA 33.4, 50, 66.7 and 100 conditions significantly depended on age (all *p*s < 0.05 except *p* = 0.741 for SOA ∞). The slope *k*, *p*-values and effect sizes (*η*^2^) are shown in the corresponding panels of Figure 4. The finding that the threshold at SOA ∞ was independent of age confirms that the target visibility was similar for observers of different ages. Therefore, the significant correlations at other SOA conditions reflect the effect of age on temporal processing.

To further reveal the differential effect of age at different SOA, we compared the slope (regression coefficients) of the linear trends across different SOA conditions. As shown in Figure 4, the slope increased as SOA increased from 16.7 to 100. The slope of linear regression at SOA 50 and SOA 66.7 was significantly steeper than that at SOA ∞ (one-tailed *t*-test, *t*(60) = 2.48, *p* = 0.011 for SOA 50; one-tailed *t*(58.4) = 2.7, *p* = 0.005 for SOA 66.7, corrected by the max-*t* procedure). These results suggest that, with increasing age, observers become more susceptible to the disturbance caused by external noise.

### Model fitting

**Figure 5:**
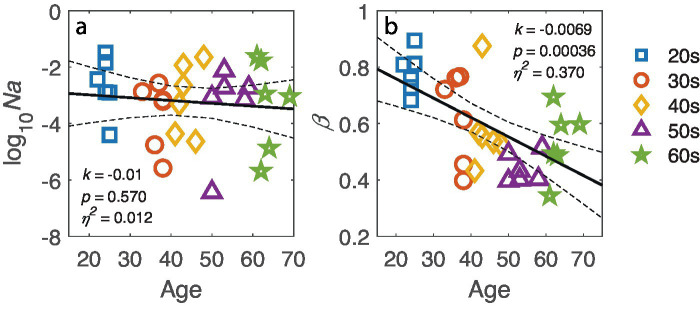
**(a)** Log-transformed internal additive noise, log10(*N*a), and **(b)** template gain *β* plotted against age. The black line represents the regression line, with dashed lines indicating 95% confidence interval. The slope *k*, *p*-value and effect size *η*^2^ are also listed in corresponding panel. Markers represent individual data points, with different colors indicating different age groups.

The ePTM provided an excellent fit to the trial-by-trial response data for all participants (all *p* > 0.05). The goodness of fit for each participant is listed in Appendix A Table A1. The model parameters *Na, Nm, β, γ, W*_16.7_*, W*_33.4_*, W*_50.0_, *W*_66.7_, and *W*_100_ were estimated from the best-fit model for each participant. Since the multiplicative noise (*N*m) and exponent (*γ*) are nuisance factors and not the primary focus of this study, only *N*a and template gain *β* are shown in Figure 5. No significant relationship was found between log internal additive noise and age (*k* = −0.01, *p* = 0.570; Figure 5a), corroborating that the target visibility was similar for observers of different ages. However, the template gain *β* significantly depended on age (*k* = −0.0069, *p* = 0.00036; Figure 5b), suggesting a decline in visual processing efficiency with increasing age.

### The temporal window throughout adulthood

ANCOVA on the temporal weights showed a significant effect of SOA [*F*(5, 140) = 233.6, *p* = 2.57 × 10^−53^]. The effect of age was not significant [*F*(1, 28) = 1.06, *p* = 0.312]. However, there was a significant interaction between age and SOA [*F*(5, 140) = 3.351, *p* = 0.012], indicating that the temporal weights at different SOAs changed with age in distinct trends.

**Figure 6:**
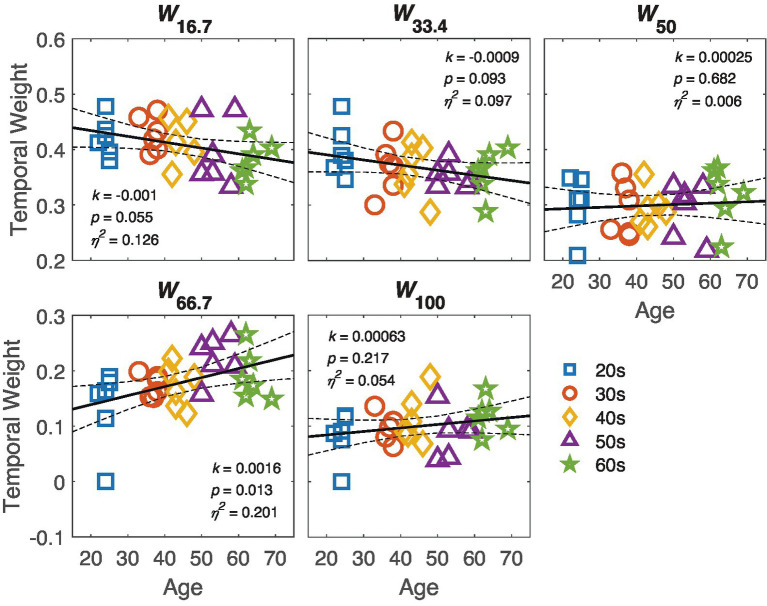
The temporal weight at different SOAs is plotted against age in the respective panels. The black line represents the regression line, with dashed lines indicating 95% confidence interval. The slope *k*, *p*-value and effect size *η*^2^ are also listed in corresponding panel. Markers indicate individual data points, with different colors indicating different age groups.

The temporal weight *W*_t_ of the perceptual template for each SOA condition is shown in the respective panels of Figure 6. The figure reveals two opposite trends: the weight at SOA 16.7 and 33.4 negatively associated with age, with marginally significance (*p*s < 0.1), while the weight at SOA 66.7 positively associated with age (*p* = 0.013). Further comparison of slopes confirmed that the slope at SOA 16.7 or SOA 33.4 was smaller than at SOA 66.7 [one-tailed *t*(58.5) = 3.32, *p* = 0.02 for SOA 16.7, and one-tailed *t*(58.8) = 3.14, *p* = 0.028 for SOA 66.7, corrected by the max-*t* procedure].

**Figure 7:**
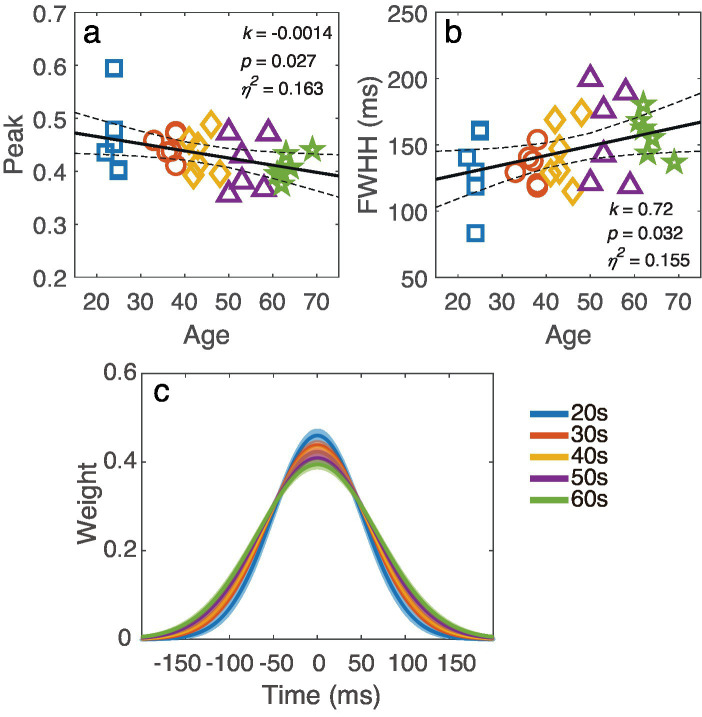
**(a)** Averaged temporal window for different decade groups, with the shaded region indicating ±1 standard error. **(b)** The peak and **(c)** FWHH of the temporal window are plotted against age. The black line represents the regression line, with dashed lines indicating 95% confidence interval. The slope *k*, p-value and effect size *η*^2^ are also listed in corresponding panel. Markers indicate individual data points, with different colors indicating different age groups.

To quantify the temporal window, a Gaussian function was fitted to the temporal weights at different SOAs. The peak amplitude and full width at half maximum (FWHM), calculated as 2 $\sqrt{2\ln\left(2\right)}\sigma$ , were derived for each observer. To investigate how the temporal window changed with age, we plotted the peak and FWHH against age in Figures 7a,b, respectively. Linear regression analysis revealed that the peak amplitude decreased as age increased (*k* = −0.0014, *p* = 0.027), while the FWHH became broader as age increased (*k* = 0.72, *p* = 0.032). To better illustrate how the temporal window changed with age, the average best-fit temporal window for each decade group is shown in Figure 7c. The results indicated that the temporal window gradually flattened with increasing age.

## Discussion

How visual processing in the temporal domain changes across adulthood is a central question in vision science. Using a dynamic external noise paradigm, we measured the contrast thresholds for orientation discrimination across various target-mask SOA conditions in observers ranging from 20 to 70 years. Our results revealed significant correlations between age and thresholds under masking conditions, but no significant correlation between age and threshold in the absence of masking. Using the ePTM model, we estimated the system’s internal additive noise (*N*a), template gain (*β*), and temporal profile *(W*_t_) from trial-by-trial response data for each observer. While no correlation was found between log *N*a and age, a significant relationship between template gain and age was observed. Further analysis of the temporal weight *(W*_t_) revealed that age influenced temporal weights at different SOAs in distinct ways. Both the peak amplitude and FWHM of the temporal window showed significant correlations with age.

The interpretation of these results hinges on two key aspects of the experimental design. First, a low spatial frequency grating was chosen as the target to control target visibility across different age groups. This was supported by the absence of a significant correlation between the contrast threshold at SOA ∞ and age, suggesting that the differential masking effect observed across age groups can be attributed solely to age-related changes in temporal processing. Second, we assumed that the masking function or temporal window is approximately symmetric around target onset. Accordingly, we used external noise masks temporally symmetricaround the target to quantify the full temporal window. This assumption is supported by prior findings showing that the shape of the masking function is approximately symmetric around zero target-mask SOA under conditions with strong masks (RMS contrast 0.33) and close temporal proximity to the target (SOA < 200 ms; Kolers, 1962; Lu et al., 2004; Breitmeyer and Öğmen, 2006).

Our finding that the masking effect was more pronounced in older adults is consistent with previous research. For instance, previous studies have shown performance deterioration in older adults (aged 59 to 83 years) compared to younger adults (aged 18 to 33 years; Atchley and Hoffman, 2004; Pilz et al., 2015; Agnew and Pilz, 2017; He et al., 2020). In a similar dynamic external noise study, He et al. (2020) found stronger masking effects in older adults compared to younger adults. Our study extends these findings by including middle-aged participants, providing further support for the notion that masking effects increase with age. The results align with those of Roinishvili et al. (2011), who found that the window of vulnerability to backward masking increased gradually across the lifespan, from 15 to 80 years.

By applying the elaborated perceptual template model (ePTM), we were able to explain the masking effect using a few key parameters of the visual system, such as template gain and the temporal window (Lu and Dosher, 1999; Lu et al., 2004; Lu and Dosher, 2008). Template gain represents the output of the observer’s template to the signal stimulus relative to its output to external noise, reflecting the overall efficiency of the visual system. We found that template gain decreased with age, consistent with our previous finding (Yan et al., 2020). By manipulating the SOA of external noise masks, we estimated the temporal weight of the perceptual template at different times, i.e., the temporal window. Older adults had lower peak amplitudes and broader temporal windows, which can explain their lower template gain and reduced efficiency. This suggests that older observers are less finely tuned to the timing of signals and are more prone to difficulties in segregating events embedded in dynamic visual inputs. The results are consistent with the findings of Busey et al. (2010) and Scurry et al. (2019). Busey et al. (2010) demonstrated age-related declines in visual temporal order judgment performance, with older adults showing reduced accuracy and higher thresholds when judging the sequence of visual stimuli, suggesting age-associated declines in multisensory integration. Similarly, Scurry et al. (2019) found that the ability to integrate and segregate multiple signals declined with age, with this impairment being consistent across visual, auditory, and cross-modal tasks. Together, these studies, along with ours, suggest an age-related impairment of the temporal integration window, in both unisensory and multisensory tasks.

Our study also provides insights into the trajectory of the temporal window across adulthood. We found that the temporal window gradually changed with age, as the peak amplitude decreased and the FWHM increased. This linear relationship between age and temporal window dimensions is consistent with McKendrick et al. (2013), who observed linear age-related changes in surround suppression. However, Roinishvili et al. (2011) found a nonlinear relationship, with masking performance remaining relatively stable until around age 50, followed by a sharp decline. This discrepancy may be due to ceiling effects in their paradigm, where the minimum measurable masking effect, represented by “vernier durations,” was 20 ms, which was already the performance limit for most younger participants. Our study, using the dynamic noise paradigm and ePTM modeling, avoids such ceiling effects and reveals a more continuous, linear pattern of change. However, the sample size of six participants per decade group was insufficient to capture the difference between the 50s and 60s age groups. The exact changes between the ages of 50 to 60, or even 70, remain to be explored in future studies.

The masking task requires the visual system to suppress irrelevant information, a process associated with inhibition in the brain. For example, using the attentional blink paradigm, Lahar et al. (2001) found that older adults struggle more than younger adults to suppress task-irrelevant information, suggesting age-related inhibitory deficits in inhibitory control (Hasher and Zacks, 1988). Similar age-related inhibitory deficits have been observed in negative priming (McDowd and Oseas-Kreger, 1991; Tipper, 1991) and Stroop effects (West and Alain, 2000). Since GABA is the main inhibitory neurotransmitter in the mammalian cortex, the age-related change in the temporal window may be due to a decrease in GABAergic inhibition (Schmolesky et al., 2000; Leventhal et al., 2003; Hua et al., 2006; Yang et al., 2008). GABA injections have been shown to improve the function of neurons in the visual cortex of old macaque monkeys (Leventhal et al., 2003). Porges et al. (2021) combined multiple datasets and found that the lifespan trajectory of cortical GABA follows an asymmetric pattern, with a rapid increase during early development, a plateau in adolescence, and a gradual decrease throughout adulthood. The change in the temporal window observed in this study across adulthood mirrors this trajectory of cortical GABA changes. Other factors might also be contributing to the age-related changes in the temporal window, as numerous structural and molecular changes occur across the lifespan. For example, a study showed that the myelination level in the human brain, which supports information processing speed, decreases across the lifespan (Bartzokis et al., 2010). How these structural and molecular factors collectively contribute to temporal processing requires further investigation.

In summary, our study found that, independent of changes in spatial processing, the temporal window of visual processing gradually flattens with age. This decline is characterized by decreasing peak amplitude and increasing FWHH. Our results suggest that the ability to process dynamic visual information deteriorates with age, with two main effects: a decrease in processing efficiency and greater vulnerability to temporal disturbances. These findings support the hypothesis that changes in functional vision occur gradually throughout adulthood.

## Data Availability

The original contributions of the study are presented in the article/supplementary material, further inquiries may be directed to the corresponding authors.

## Appendix A

**Table A1:** Goodness of fit of the ePTM.

Participant no.	Age	*X* ^2^	*p*
1	22	242.8	0.999489
2	24	246.26	0.998877
3	24	254.34	0.994294
4	24	238.49	0.999823
5	25	244.46	0.999248
6	25	226.15	0.999995
7	33	243.5	0.999397
8	36	228.63	0.999989
9	37	227.31	0.999993
10	38	243.36	0.999417
11	38	242.65	0.999506
12	38	229.23	0.999987
13	41	226.98	0.999993
14	42	244.11	0.999306
15	43	245.53	0.999043
16	43	250.11	0.99747
17	46	253.41	0.995191
18	48	252.42	0.996014
19	50	241.15	0.999655
20	50	249.02	0.997978
21	53	247.62	0.998491
22	53	242.09	0.999568
23	58	255.86	0.992492
24	59	248.96	0.998
25	61	264.95	0.968267
26	62	238.42	0.999826
27	62	254.87	0.99371
28	63	223.68	0.999998
29	64	229.25	0.999987
30	69	240.58	0.9997
